# Epigenetic modifications in esophageal cancer: An evolving biomarker

**DOI:** 10.3389/fgene.2022.1087479

**Published:** 2023-01-10

**Authors:** Wen-Jian Liu, Yuan Zhao, Xu Chen, Man-Li Miao, Ren-Quan Zhang

**Affiliations:** ^1^ Department of Thoracic Surgery, First Affiliated Hospital of Anhui Medical University, Hefei, Anhui, China; ^2^ School of Basic Medicine, First Affiliated Hospital of Anhui Medical University, Hefei, Anhui, China

**Keywords:** esophageal cancer, epigenetic modifications, biomarker, DNA methylation, anti-cancer therapy

## Abstract

Esophageal cancer is a widespread cancer of the digestive system that has two main subtypes: esophageal squamous cell carcinoma (ESCC) and esophageal adenocarcinoma (EA). In the diverse range of cancer therapy schemes, the side effects of conventional treatments remain an urgent challenge to be addressed. Therefore, the pursuit of novel drugs with multiple targets, good efficacy, low side effects, and low cost has become a hot research topic in anticancer therapy. Based on this, epigenetics offers an attractive target for the treatment of esophageal cancer, where major mechanisms such as DNA methylation, histone modifications, non-coding RNA regulation, chromatin remodelling and nucleosome localization offer new opportunities for the prevention and treatment of esophageal cancer. Recently, research on epigenetics has remained at a high level of enthusiasm, focusing mainly on translating the basic research into the clinical setting and transforming epigenetic alterations into targets for cancer screening and detection in the clinic. With the increasing emergence of tumour epigenetic markers and antitumor epigenetic drugs, there are also more possibilities for anti-esophageal cancer treatment. This paper focuses on esophageal cancer and epigenetic modifications, with the aim of unravelling the close link between them to facilitate precise and personalized treatment of esophageal cancer.

## 1 Introduction

Esophageal cancer is a prevalent gastrointestinal tumour, a malignant tumour occurring in the mucosal epithelium of the oesophagus, that can be divided into two main categories: esophageal squamous cell carcinoma (ESCC) and esophageal adenocarcinoma. Of these, the former is predominant in clinical practice (Short et al., 2017). The latest cancer data show a steady increase in the overall 5-year relative survival rate for all cancers, but the survival rate for esophageal cancer is at the bottom of the list at only 20% (Siegel et al., 2022). The latest estimate from GLOBOCAN 2020 for esophageal cancer is 6.3 cases and 5.6 deaths per 100,000 people in 2020 globally. Further projections suggest that there will be 957,000 new cases and 880,000 deaths from esophageal cancer by 2040 (Morgan et al., 2022). Surgery, the main curative treatment for early-stage localized esophageal cancer, has limitations because it does not provide a standard of care for patients with distant metastases in the mid to late stages. Furthermore, some of the side effects and high costs associated with radiotherapy remain an unbridgeable gap. Hence, there is an urgent need to find new drugs with multiple targets, good efficacy, few side effects and low cost.

Cancer has a variety of biological features, including cell differentiation, abnormal proliferation, infiltration, and metastasis. Its occurrence involves a complex multifactorial, multistep process and is closely related to smoking, infection, occupational exposure, environmental pollution, irrational diet and genetic factors (Mullard, 2020). In recent years, there has been a surge of research into epigenetics, with a focus on translating the basic into the clinical setting and transforming epigenetic alterations into targets for cancer screening and detection in the clinic. Numerous studies have been performed to this end. Epigenetics is the opposite concept to genetics, which is a science based on changes in gene expression levels due to non-genetic sequence alterations, whose mechanisms involve DNA methylation, histone modifications, non-coding RNA regulation, chromatin remodelling and nucleosome localization. Studies have also confirmed that abnormal epigenetic mechanisms affect the transcription of genes that are widely involved in cell growth, differentiation, apoptosis, transformation and tumour progression, all of which have valuable implications for the clinical diagnosis, treatment and prevention of tumours (Sun et al., 2022). In addition, most cancers share an essential feature, which is the presence of DNA alterations in the genome and epigenome. Cancer cells can acquire genetic and epigenetic alterations that alter the molecular and cell biological processes of the cell that drive cancer initiation and progression. These alterations, in addition to host and other environmental factors, ultimately lead to the clinical behaviour of cancer. The promising point is that these alterations can be used in the clinic as biomarkers for researchers to determine cancer risk, for early detection of cancer and precancer, to determine cancer prognosis and to predict response to treatment. Of note, epigenetic regulatory mechanisms play a very important role in the development of esophageal cancer. This article reviews the progress of epigenetic regulatory mechanisms in esophageal cancer.

## 2 DNA methylation and esophageal cancer

Currently, nothing is more vigorously researched than epigenetic modification, which has greatly contributed to the development of molecular genetics. In turn, DNA methylation is the most well-studied form of epigenetic modification. Studies have shown that normal methylation is necessary to maintain cell growth and metabolism, while abnormal DNA methylation can cause disease, such as tumours (Nishiyama and Nakanishi, 2021). Therefore, the study of DNA methylation is very useful for understanding biological growth and development as well as disease treatment.

The methylation status of DNA not only affects the growth and development of an organism but also plays an important role in the development of tumours. The cancer process in esophageal cancer can be broadly understood as a period of cellular genetic mutation, a period of precancerous lesions and a period of clinical cancer. Within this, how DNA methylation is involved and regulated is the focus of what researchers want to determine. DNA methylation is known to occur on CpG islands, which are often located near transcriptional regulatory regions. Studies have shown that the DNA methylation that occurs here is highly correlated with the carcinogenesis of esophageal cancer. Specifically, during the carcinogenesis of esophageal cancer, altered DNA methylation status in CpG islands can lead to increased chromosomal rotation and silencing and loss of expression of oncogenes, leading to tumour growth (Xi et al., 2022). A variety of related gene methylations have been examined in esophageal cancer, such as p16, E-cadherin, DNMT1 and MTHFR.

The P16 gene, also known as the multiple tumour suppressor 1 (MTS1) gene, was a new anticancer gene discovered by Kamb et al., in 1994 and is a fundamental gene in the cell cycle, that is directly involved in the regulation of the cell cycle, negatively regulating cell proliferation and division (Kamb et al., 1994). Once inactivated, it causes malignant cell proliferation. Numerous studies have corroborated this. The P16 gene was found to be widely involved in the formation of a variety of tumours by gene deletion and mutation. Detecting the presence or absence of alterations in the p16 gene is of great clinical importance in determining the susceptibility of patients to tumours and in predicting the prognosis of tumours (Foulkes et al., 1997; Zhao et al., 2016). Hibi et al. (2001) applied methylation-specific PCR to detect serum from 38 patients with ESCC and found that the rate of p16 methylation was 82%. Bian et al. (2002) examined the degree of P16 methylation in tissue specimens from 22 esophageal adenocarcinoma and 33 precancerous lesions, of which P16 methylation was detected in 18 and 10 cases with methylation rates of 82% and 30%, respectively. A study by Guo et al. (2006) further showed that P16 methylation was most common in ESCC, at approximately 52%. Further additions were made by Li et al. (2011) They suggested that the pathogenesis of ESCC was associated with hypermethylation of several tumour-related genes, such as RAR-β, DAPK, p16 and CDH1, mediated by increased expression of DNMT3b. Of note, genistein and other isoflavones from soy can reactivate methylation-silenced genes, such as P16, and inhibit the growth of esophageal cancer cells, in part by directly inhibiting DNA methyltransferases (Fang et al., 2005).

E-cadherin, a transmembrane glycoprotein with a molecular weight of 120 kDa, belongs to a family of calcium-dependent adhesion molecules in epithelial cells and plays an influential role in the growth and development of tissues. The reduction or loss of its function can lead to the disruption of cell junctions and is associated with the infiltration and metastasis of tumour cells. Corn et al. (2001) examined tissue specimens of esophageal adenocarcinoma by MSP and immunohistochemistry and found that the positive rate of E-cadherin methylation was 84% in 31 samples and that immunohistology suggested that E-cadherin protein expression levels were significantly lower in methylated samples than in non-methylated samples. This was also confirmed in a study by Si et al. (2001) The hypermethylation rate was 80% in 20 specimens of ESCC. In addition, Lee et al. (2008) retrospectively analysed the methylation status of 251 cases of ESCC, and the E-cadherin detection rate reached 43%. Hypermethylation of the E-cadherin gene and integrin alpha4 gene can be used as prognostic indicators related to recurrence of stage I and II ESCC, respectively.

The process of DNA methylation is dependent on the function of DNA methylation enzymes (DNMTs). DNMTs are aberrantly expressed in a variety of diseases, and their expression is particularly elevated in many malignancies. DNMT1 is one of the most extensively studied isoforms of the DNMT family and is the most abundant methyltransferase in human cells. It maintains DNA methylation, and its aberrant expression destabilizes the genome, playing an important role in the development and progression of many diseases (Fu et al., 2022). Seiji et al. observed the effect on drug-induced squamous carcinoma of the tongue and oesophagus in mice by shaping the DNA hypomethylation state using the DNMT1 allele. Their results showed that the DNA hypomethylation state significantly inhibited squamous carcinogenesis of the tongue and oesophagus (Baba et al., 2009). Similarly, only 5% black raspberries significantly inhibited drug-induced ESCC in mice. They also further confirmed that this inhibition was associated with reduced mRNA levels of DNMT1 and DNMT3b (Huang C et al., 2016). In addition, some miRNAs, as well as the combined regulatory mechanism of LncRNA and DNMT1, have been suggested as novel mechanisms for the development of esophageal carcinogenesis (Li et al., 2014; Zeng W et al., 2019).

Methylenetetrahydrofolate reductase (MTHFR) is the rate-limiting enzyme that regulates the metabolism of folate and methionine. It can reduce 5,10-methylenetetrahydrofolate to 5-tetrahydrofolate, thus acting as an indirect donor of methyl groups to participate in the synthesis of purines and pyrimidines and the methylation of DNA, RNA, and proteins *in vivo* while maintaining the effectiveness of the normal peer cysteine cycle *in vivo* and ensuring the proper functioning of DNA synthesis and methylation. Song et al. designed an experiment to investigate the MTHFR gene polymorphism and the risk of ESCC in northern China. The obtained results showed that genetic polymorphisms in the MTHFR gene may be a predisposing factor for ESCC in an experimental group of 240 patients with esophageal cancer and a control group of 360 healthy individuals (Song et al., 2001). It has also been demonstrated that aberrant DNA methylation in the genetic polymorphisms of the P16, MGMT and hMLH1 genes and MTHFR C677T in ESCC is significantly associated and is most likely a promising biomarker for diagnosis and prognosis (Wang et al., 2008).

In addition, GPX3, EYA4, PAX1, SOX1, ZNF582, SLC22A3, and Polι, all of which were implicated in DNA methylation in esophageal cancer, which in turn influenced the increase or decrease in their expression, could be key factors in the carcinogenesis of esophageal cancer (Lin et al., 2020; Wang H et al., 2021). The details are shown in Table 1.

**TABLE 1:** Biomarkers of abnormal DNA methylation in esophageal carcinoma.

Name	Function	Methylation status	Ref
p16	Involved in the regulation of cell cycle	Hypermethylation status	Li et al. (2011)
E-cadherin	Involved in the regulation Epithelial-mesenchymal transition	Hypermethylation status	Ling et al. (2011)
DNMT1	Maintenance methylation enzymes	Hypermethylation status	Zeng W et al. (2019)
MTHFR	Involved in cell cycle regulation, DNA replication, DNA and protein methylation modifications	Significantly associated with hypermethylation status	Wang et al. (2008)
GPX3	Abnormal expression in a variety of tumors	Hypermethylation status	He et al. (2011)
EYA4	Involved in apoptosis regulation, innate immunity, DNA damage repair, angiogenesis	Hypermethylation status	Luo et al. (2018)
PAX1	Involved in regulation of transcription, DNA dependence, promoter development	Hypermethylation status	Tang et al. (2019)
SOX1	Involved in establishing and maintaining chromatin structure, regulating transcription, DNA-dependent	Hypermethylation status	Tang et al. (2019)
ZNF582	Involved in transcriptional regulation	Hypermethylation status	Tang et al. (2019)
SLC22A3	Mediates potential-dependent transport of a variety of organic cations	Hypermethylation status	Xiong et al. (2018)
Polι	Involved in the translation and synthesis of DNA	Hypomethylation state	Zhou et al. (2012)

## 3 Histone modifications and esophageal cancer

In the mammalian genome, histones can be modified in many forms by the action of related enzymes, such as methylation, acetylation, phosphorylation, adenylation, ubiquitination and ADP-ribosylation, all of which affect the transcriptional activity of genes (Audia and Campbell, 2016). Therefore, a proper understanding of the role of histone modifications in the development of cancer is an essential part of the process.

Furthermore, in addition to DNA methylation, histone modification is also an important event in human esophageal cancer pathology, and histone methylation and acetylation are also important modes of gene expression regulation. Histone methylation sites are mostly located on lysine and arginine residues of H3 and H4 and are dynamically regulated by histone arginine methyltransferases and lysine demethylases, which can regulate gene expression and maintain chromatin structure at the corresponding sites. Cao et al. (2020) used a whole methylome view to map out the oncogenic factors of ESCC. The findings revealed that 98% of CpG was methylated in the entire ESCC genome and was enriched in the H3K9me3 and H3K27me3 regions. Zhao et al. (2019) found that PRMT1 could promote the transcriptional activation of downstream genes, mainly by catalysing asymmetric demethylation of histone H4R3. The mechanisms involved in the activation of Wnt/β-catenin and Notch signalling pathways were further explored by RNA-Seq transcriptome analysis. In addition, H3K9me3, H4K20me3 and H3K36me3 have been suggested to be closely associated with clinical features and are independent risk factors for patients with esophageal cancer. Zhou et al. (2019) even further suggested that the combination of the three expressions could be expected to further enhance the assessment of prognosis and management of esophageal cancer.

Histone acetylation is a reversible, biologically dynamic process regulated by the dual regulation of histone acetyltransferase (HAT) and histone deacetyltransferase (HDAC). The dynamic balance of acetylation and deacetylation also affects chromatin structure and gene expression, selectively affecting the structure of chromatin regions and thus gene expression. In addition, HAT and HDAC play a crucial role in the regulation of chromatin function and cell death, and their misregulation may be associated with the development of certain tumours in humans, as demonstrated by Toh et al. (2003) in a study of esophageal cancer. In addition, histone acetylation has been considered a potential novel chemotherapeutic target to inhibit cancer cell proliferation. Inhibitors developed for this purpose are also considered to be promising anticancer agents. Ahrens et al. (2015) showed that the combination of HDAC inhibitors (HDACi) and azacytidine could selectively target esophageal cancer by inducing DNA damage, inhibiting cell viability, and inducing apoptosis. Hu et al. (2015) first found that methylseleninic acid could induce acetylation of histone H3 at Lys9, thereby inhibiting the growth of esophageal cancer cells. The mechanism also involved the HAT/HDAC interaction. In addition, aberrant histone modifications often accompany and interact with DNA methylation, further complicating the mechanisms of gene expression in tumour cells (Chang et al., 2019). Based on immunohistochemistry and other experiments, Tzao et al. (2006) found a high degree of concordance between promoter methylation of the Fragile histidine triad (FHIT) and aberrant expression of FHIT and acetylated H4, with specific concordance rates of 75% and 81.7%, respectively.

Relatively speaking, histone methylation modifications are the most stable and therefore best suited as stable epigenetic information, while acetylation modifications have a higher dynamic. There are also other unstable modifications, such as phosphorylation, adenylation, ubiquitination and ADP-ribosylation, which are more flexible and can affect the structure and function of chromatin and thus perform their regulatory functions. The combinatorial variations between these histone modifications are so numerous that histone covalent modifications may be a more refined form of gene expression (Strahl and Allis, 2000).

## 4 Non-coding RNA regulation and esophageal cancer

In recent years, with the development of molecular biology, whole genome sequencing analysis has identified an increasing number of non-coding RNAs (ncRNAs) that play an important role in tumour development, infiltration and metastasis (Anastasiadou et al., 2018). There are two main categories of ncRNAs, structural ncRNAs, mainly rRNA and tRNA. The other category is regulatory ncRNAs, which are classified into small, medium, and long ncRNAs according to their nucleotide length. Small ncRNAs are between 20 and 50 nucleotides in length, with miRNAs and siRNAs being the most widely studied. Medium ncRNAs are 50–200 nucleotides in length and include snoRNA and snRNA. lncRNAs are greater than 200 nucleotides in length. Studies have shown that lncRNAs play important roles in many life activities, such as epigenetic regulation, cell cycle regulation and cell differentiation regulation, and have become a hot spot in genetic research (Bhan et al., 2017).

**FIGURE 1:**
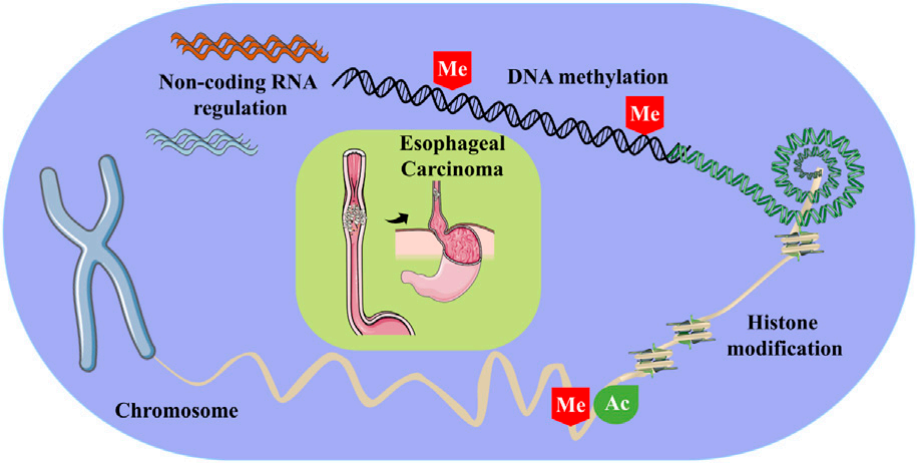
Some of the epigenetic modifications involved in esophageal carcinoma, including DNA methylation, histone modifications and non-coding RNA regulation. Of these, Me stands for methylation, and Ac stands for acetylation.

NcRNAs can act not only in the diagnosis of esophageal cancer but also in the treatment and prognosis of esophageal cancer. Liu A et al., 2017 used miR-455-3p antagonists and found that they could greatly improve the sensitivity of chemotherapy in esophageal cancer. The newly identified miR-99b/let-7e/miR-125a cluster also plays an important role in tumour metastasis, and its overexpression promotes the migration and invasion of esophageal cancer cells *in vitro* and *in vivo* (Ma et al., 2017). miR-143 and miR-145 also have the potential to act as common anticancer substances in esophageal cancer (Wu et al., 2011). In addition, studies have shown that miR-3656, -498, -32, -375 and -27b-3p can be used as novel diagnostic and prognostic biomarkers for esophageal cancer and have strong potential (Islam et al., 2017; Liu Y. T et al., 2019; Han et al., 2020; Jin et al., 2021; Wu et al., 2021). In addition, lncRNAs have equal potential; for example, Xue et al. (2022) used transcriptome analysis and found that LINC00680 was highly expressed in esophageal cancer and significantly correlated with tumour volume, stage and prognosis. Further experiments revealed that inhibition of LINC00680 expression could inhibit the proliferative properties of esophageal cancer *in vitro* and *in vivo* to some extent. The mechanism is mainly that LINC00680 can act as a ceRNA that can sponge miR-423-5p, which in turn regulates the expression of p21-activated kinase 6 (PAK6) in esophageal cancer cells. The exosomal lncRNA ZFAS1 can regulate STAT3 and miR-124 to promote the proliferation, migration and invasion of esophageal cancer cells and inhibit their apoptosis, which leads to tumour progression (Li et al., 2019). The lncRNA MIR22HG has also been suggested as a novel cancer prognostic biomarker acting through ceRNA (Zhang C et al., 2020). Similarly, the lncRNAs NORAD, AGPG, DNM3OS, VESTAR, IRF1-AS and LINC01554 have strong potential for application in the diagnosis and treatment of esophageal cancer (Zhang B. X et al., 2019; Huang et al., 2019; Liu Y et al., 2020; Wang Y et al., 2021; Jia et al., 2021; Zheng et al., 2022) (Figure 1; Table 2).

**TABLE 2:** Biomarkers of ncRNAs in esophageal carcinoma.

ncRNAs	Expression	Mechanism	Ref
lncRNA CASC9	Upregulated	Interacts with CREB-binding proteins to upregulate LAMC2 expression and promote metastasis	Liang et al. (2018)
LncRNA TUG1	Upregulated	Regulation of the miR-1294/PLK1 axis, an oncogene for esophageal cancer	Zong et al. (2020)
LncRNA ELFN1-AS1	Upregulated	Acts as a ceRNA to promote progression by sponging miR-183-3p, which promotes GFPT1 expression	Zhang H et al. (2020)
LncRNA FAM83A-AS1	Upregulated	Exacerbation of malignant development through negative regulation of miR-495-3p	Huang et al. (2020)
LncRNA RPL34-AS1	Downregulated	Inhibition of cell proliferation, migration and invasion by down-regulation of RPL34 expression	Gong et al. (2019)
lncRNA ZFAS1	Upregulated	Up-regulation of STAT3 and down-regulation of miR-124 promote cell proliferation, migration and invasion and inhibit apoptosis	Li et al. (2019)
LncRNA SNHG7	Upregulated	Regulates the expression of p15 and p16, partially contributing to development	Xu L. J et al. (2018)
LncRNA linc00460	Upregulated	Acts as a molecular sponge to adsorb miR-1224-5p, which promotes cell migration, invasion and EMT	Cui et al. (2020)
LncRNA MIR22HG	Downregulated	Functions as ceRNA, participates in signaling pathways, interacts with proteins/miRNAs and acts as a host gene in tumorigenesis and tumor progression	Zhang C et al. (2020)
LncRNA EIF3J-AS1	Upregulated	Demonstrates oncogenic properties by acting as a sponge for miR-373-3p to upregulate AKT1 mRNA levels	Wei et al. (2020)
miR-485-5p	Upregulated	Significantly inhibits cell proliferation, migration and invasion	Han et al. (2019)
miR-498	Downregulated	Inhibition of autophagy and M2-like polarization of macrophages through MDM2/ATF3, leading to cancer inhibition	Li et al. (2021)
miR-502	Upregulated	Promotes phosphorylation of AKT signaling and regulates cell proliferation	Xu J et al. (2018)
miR-493	Downregulated	Inhibits c-JUN and p-PI3K/p-AKT activity, enhances p21, and directly regulates the expression and function of Wnt5A	Bian et al. (2021)
miR-2053	Downregulated	Upregulation of KIF3C expression and activation of PI3K/AKT signaling pathway involved in cell proliferation, apoptosis, migration and invasion	Yao et al. (2021)
miR-183	Upregulated	Regulation of PDCD4 expression, which acts as a carcinogen	Yang et al. (2014)
miR-4885	Upregulated	Binds to the 3′untranslated region of CTNNA2, reduces cell adhesion and promotes EMT	Song et al. (2019)
miR-374a	Upregulated	Reduced expression and transcriptional activity of Axin2 and inhibition of cell proliferation	Wang et al. (2015)
miR-483-5p	Upregulated	Targeted silencing of KCNQ1 as an oncogene promotes cell proliferation, migration and invasion	Chen et al. (2020)
miR-10b	Upregulated	Targeting PPARγ to activate AKT/mTOR/P70S6K signaling and confer cisplatin resistance	Wu K et al. (2020)

## 5 Others

In addition to the abovementioned epigenetic modifications involved in the progression of esophageal cancer, chromatin remodelling and nucleosome localization are also involved in the development of esophageal cancer as important epigenetic mechanisms. Sung et al. (2015) analysed and examined the risk assessment of epigenetic modifications and upper gastrointestinal tract tumours using thousands of cases, and it is certain that chromatin remodelling occupies an important place in cancer risk. The SWI/SNF complex is an important chromatin remodelling factor, and on this basis, Schallenberg et al. reported the loss of SWI/SNF enzyme subunits in a large number of esophageal cancers. This reflects the important role of chromatin remodelling in esophageal cancer. In addition, nucleosome localization is an essential mechanism that regulates transcription by inhibiting or facilitating the binding of transcription factors. The stability of nucleosome localization may even be considered a regulator of variation in germline mutation rates in the human genome (Li and Luscombe, 2020). Currently, there is less focus on the link between esophageal cancer and nucleosomes, but it is no less an area of research with important implications for human genetics and genome evolution.

## 6 Epigenetically linked pathways in esophageal cancer

Cancer-related signalling pathways play a crucial role in regulating cellular processes. Furthermore, epigenetic modifications, which are currently a hot topic of research, still need to be investigated in depth. Therefore, the study of the specific mechanisms and functions between them will provide us with a deeper understanding of cancer-related signalling pathways and provide valuable information for new drugs to treat cancer.

PI3K/AKT/mTOR, as the star molecular pathway in cancer-related pathways, has been found in various tumours, including esophageal cancer. Huang et al. demonstrated that the PI3K/AKT/mTOR signalling pathway is a potential therapeutic target for ESCC by summarizing the extensive literature (Huang et al., 2022). Luo et al. (2022) also confirmed this to a certain extent. In addition, miR-18a can increase the expression of chylin D1 by regulating the PTEN-PI3K-AKT-mTOR signalling axis, thus promoting the proliferation of esophageal cancer Eca109 cells (Zhang et al., 2016). miR-214, as a tumour promoter, targets LZTS1 through the PI3K/AKT/mTOR signalling pathway to promote the proliferation, migration, invasion and inhibition of apoptosis of ESCC cells (Guanen et al., 2018). The dual PI3K-HDAC inhibitor CUDC-907 inhibits the PI3K-AKT-mTOR pathway, leading to the downregulation of LCN2 expression and ultimately to the accumulation of ROS and the activation of cytotoxic autophagy. In addition, consistent antitumor effects have been demonstrated in xenograft mouse models (Jian et al., 2022).

The MAPK/ERK pathway is one of the branches of the MAPK pathway, which also includes JNK, p38/MAPK and ERK5. JNK, and p38 have similar functions and are related to inflammation, apoptosis and growth, while ERK is mainly responsible for cell growth and differentiation. Zhang H et al. (2019) showed that miR-148a was significantly downregulated in ESCC and predicted a poor prognosis for patients. They further demonstrated that miR-148a directly targets its target gene MAP3K9 and affects the ERK/MAPK pathway and EMT-related pathways in ESCC, inhibiting cell proliferation and invasion. Overexpression of miR-133b has also been shown to target EGFR, thereby inhibiting the MAPK/ERK and PI3K/AKT signalling pathways and suppressing cell proliferation, migration and invasion (Zeng B et al., 2019). KISS-1, a tumour suppressor mediated by promoter methylation, downregulates the expression of phosphorylated ERK1/2 and MAPK to inhibit the metastasis of ESCC cells by targeting the MMP2/9/ERK/p38 MAPK axis (Duan et al., 2022).

The Wnt/β-catenin signalling pathway has been found to be aberrantly activated in a variety of tumours. It has been demonstrated that inhibition of Wnt/β-catenin signalling increases the sensitivity of esophageal cancer cells to radiotherapy (Spitzner et al., 2021). Cao et al. (2020) used a high-resolution multiomics approach to map the epigenetic landscape of ESCC, suggesting that the epigenetically mediated Wnt/β-catenin signalling pathway is a potential oncogenic driver of ESCC. Li et al. (2010) found that Wnt5A, which is often silenced by promoter methylation in ESCC, could suppress tumours by antagonizing the Wnt/β-catenin pathway. In addition, the epigenetic modifier PRDM5 was shown to act as a tumour suppressor by regulating the Wnt/β-catenin signalling pathway through a mechanism involving antagonism with aberrant Wnt/β-catenin signalling and inhibition of oncogene expression (Shu et al., 2011).

**FIGURE 2:**
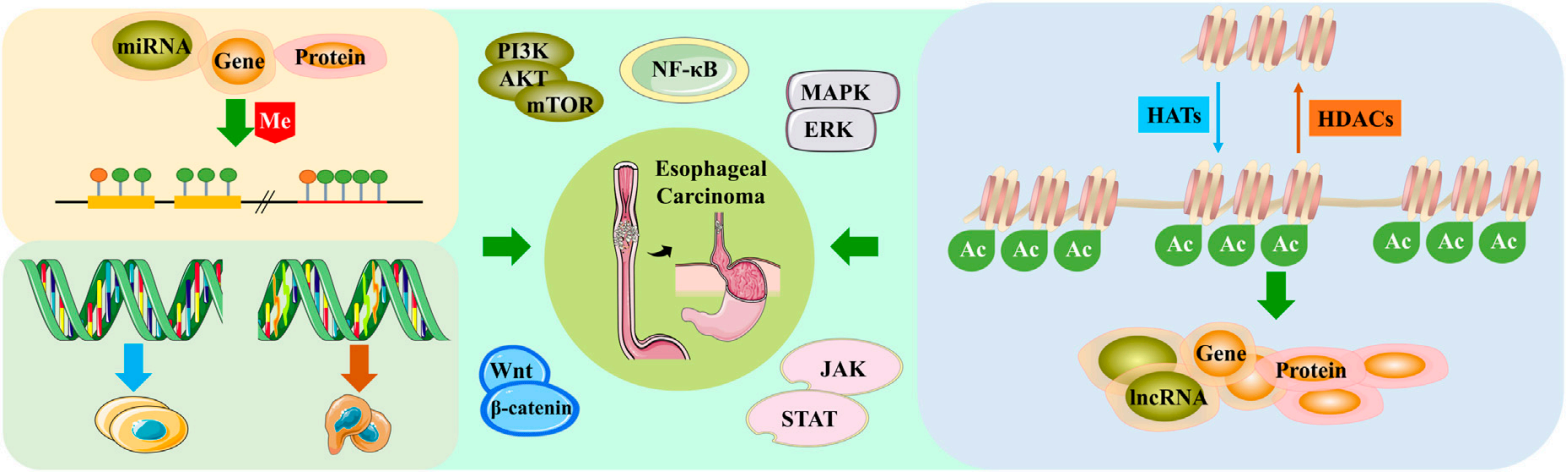
Esophageal cancer regulates cancer-related pathways through different epigenetic modifications.

In addition, the NF-κB and JAK-STAT pathways are also involved. Studies have shown that hypomethylation-induced PLCE1 can induce angiogenesis and inhibit apoptosis through activation of the NF-κB signalling pathway (Chen et al., 2019). miR-429 inhibits proliferation in ESCC *via* the RAB23/NF-κB pathway (Wang S et al., 2020). Similarly, JAK-STAT structural domain-enhanced MUC1-CAR-T cells exhibit significant anti-esophageal cancer potential *in vivo* (Zhang L et al., 2020). miR-193, on the other hand, is thought to enable esophageal cancer cells to acquire cisplatin resistance by regulating the cell cycle (Shi et al., 2020) (Figure 2).

## 7 Epigenetics in the treatment of esophageal cancer

According to the latest cancer data, people are still under constant threat from cancer. Cancer not only affects the patient but also has economic and social implications. It is imperative for patients to receive active and effective treatment. In addition, the choice of the right treatment can also have a significant impact on a patient’s life and health. In addition to surgery, radiotherapy and chemotherapy, new immunotherapies and gene therapies are being actively developed that can treat cancer by inhibiting and killing tumour cells either directly or indirectly. Among these, epigenetics has emerged as a research hotspot, and by virtue of its reversible response, a range of epigenetic drugs have been developed, such as DNA methyltransferase inhibitors (DNMTi) and HDACi, to treat disease by altering DNA methylation and histone modification patterns. Nutlin-3, as a DNMTi, greatly improves the sensitivity of esophageal cancer to radiotherapy (Chang et al., 2022). Ahrens et al. (2015) combined HDACi with azacytidine to treat esophageal cancer cells and found that they could modulate some of the novel candidate genes in esophageal cancer, which may be effective in treating esophageal cancer. Bruyer et al. (2018) combined the two and found them to be more beneficial for patients at high risk of multiple myeloma. In addition, they have clear therapeutic value in colorectal cancer, breast cancer and melanoma (Raynal et al., 2017; Gonçalves et al., 2020; Grunewald et al., 2021).

To investigate the clinical value of epigenetic regulation in esophageal cancer, Liu J et al. (2020) found that pretreatment of esophageal cancer cells with decitabine, a DNMTi, reversed the methylation of IGFBPL1, thereby regulating the PI3K-AKT signalling pathway to inhibit esophageal cancer proliferation *in vitro* and *in vivo*. Decitabine can also enhance the recognition of esophageal cancer by T cells through upregulation of MAGE-A3 expression, which can contribute to further immunotherapy (Shi et al., 2019). Low doses of decitabine also inhibited the invasive ability of esophageal cancer cells (Liu et al., 2014). In addition, the combination of decitabine and azacytidine also contributes to the suppression of esophageal cancer activity (Ahrens et al., 2015). Procaine, a non-nucleoside DNMTi, can inhibit the proliferation of gastric cancer cells (Li et al., 2018) but has not been reported in esophageal cancer. Another inhibitor derived from green tea, (-)-epigallocatechin-3-gallate, can inhibit the proliferation of esophageal cancer (Wang et al., 2019). In addition, it can also inhibit esophageal cancer by promoting apoptosis and reversing multidrug resistance (Liu L et al., 2017). HDACi is also featured in epigenetic drug development, such as FK228, entinostat and belinostat, which are in trials. Hoshino et al. (2005) found that FK228 could activate peroxiredoxin 1 expression and regulate histone acetylation in the promoter to achieve an anti-esophageal cancer effect. This was confirmed by subsequent analysis of the gene expression profile of esophageal cancer (Hoshino et al., 2007). Entinostat has been demonstrated to overcome cisplatin resistance in esophageal cancer cells by a mechanism involving the Src-Mcl-1-MDR1 pathway (Huang et al., 2018), and it also induces DNA damage and apoptosis in esophageal cancer (Feingold et al., 2018). In contrast, Belinostat has been used to treat peripheral T-cell lymphoma (O’Connor et al., 2015). It has not been reported in esophageal cancer. Furthermore, there have been a number of clinical trials that have further validated the efficacy of some of the abovementioned drugs in the treatment of esophageal cancer (NCT00423150, NCT01799083, NCT01386346, NCT02625623) (Hochhauser et al., 2013; Schneider et al., 2017; Bang et al., 2018; Chen et al., 2018). In addition, miRNAs are an important target for epigenetic therapy. It has been shown that the development of esophageal cancer can be inhibited by reducing the expression of oncogenic miRNAs, increasing the expression of oncogenic miRNAs and interfering with miRNA‒mRNA interactions (Sharma and Sharma, 2015). In addition, because epigenetic abnormalities are not single isolated events, they are interlinked with each other. Hence, epigenetics-based combination therapeutic strategies may also further improve the outcome of esophageal cancer treatment (Nasir et al., 2020).

In addition to the epigenetic drugs that have been developed thus far, some herbal active ingredients can also modulate epigenetic modifications to achieve antitumor effects. Studies have shown that resveratrol, a powerful alternative to antioxidants, inhibits a variety of cancers (Rauf et al., 2018). Luteolin inhibits paclitaxel resistance in esophageal cancer and synergistically inhibits the EMT process in combination with low-dose paclitaxel, thereby inducing apoptosis in esophageal cancer *in vitro* and *in vivo* (Zhao Y et al., 2021; Qin et al., 2021). In addition, apigenin, icaritin, oridonin, berberine and curcumin have all been shown to have anti-esophageal cancer efficacy (Jiang et al., 2017; Han et al., 2018; Jiang et al., 2019; Kwiecien et al., 2019; Qiu et al., 2019). The modulation of epigenetic modifications by traditional Chinese medicine (TCM) partially explains the antitumor mechanism of TCM and may not be the only mechanism to be further investigated.

## 8 Epigenetics in the prognosis of esophageal cancer

The formation of cancer requires a combination of factors, including abnormal genetic alterations, including the activation of proto-oncogenes, inactivation of oncogenes, accumulation of multiple genetic abnormalities, and involvement of pathogenic factors, ultimately leading to the formation of cancer. The treatment of cancer mostly adopts a combination of surgery, chemotherapy, and radiotherapy and can be combined with targeted therapy and biological therapy if needed to improve the prognosis. Cancer has long been one of the most important diseases threatening human life and health. Early-stage patients may be cured with timely and effective treatment, while patients in the middle and late stages are more difficult to cure, with high recurrence and metastasis rates, but active treatment can delay the progression of the disease and improve survival rates. Epigenetic modifications are widely involved in the development of tumour diseases and have played an important role in the early diagnosis, prognosis evaluation and development of therapeutic drugs in recent years.

DNA methylation was one of the first epigenetic modifications to be identified and the most intensively studied to date. In clinical applications, the detection of methylation of specific genes in serum, urine, or tissue fluids for early diagnosis of disease has the advantage of being convenient, rapid, and non-invasive and is also highly specific and sensitive. Xi et al. (2022) confirmed the important role of aberrant DNA methylation in the development and progression of esophageal cancer by comparing 91 genome-wide methylation sites in esophageal cancer and adjacent normal tissues that matched them, laying the foundation for the future development of non-invasive cancer detection methods for targeted methylation testing. Talukdar et al. (2021) studied cases of esophageal cancer from nine high incidence countries in Africa, Asia and South America, focusing on aberrant DNA methylation in them, suggesting that it could serve as a potential tumour-specific marker for esophageal cancer for prognosis and even treatment. In another study, hypermethylation of the promoter region of the APC gene was observed in 48 of 52 patients with esophageal adenocarcinoma and in 16 of 32 patients with squamous esophageal carcinoma, with methylation rates of approximately 92% and 50%, respectively. Hypermethylated APC DNA was also detected in patient plasma and was significantly associated with patient survival. Kawakami et al. (2000) suggested that this may be a strong prognostic marker for esophageal cancer. Smoking and alcohol consumption are risk factors for esophageal cancer, which can induce methylation changes and are involved in cancer-related pathways through multiple pathways and genes. The characterization of the DNA methylome will help to better understand its mechanisms and improve its prognosis (Ma et al., 2016).

**FIGURE 3:**
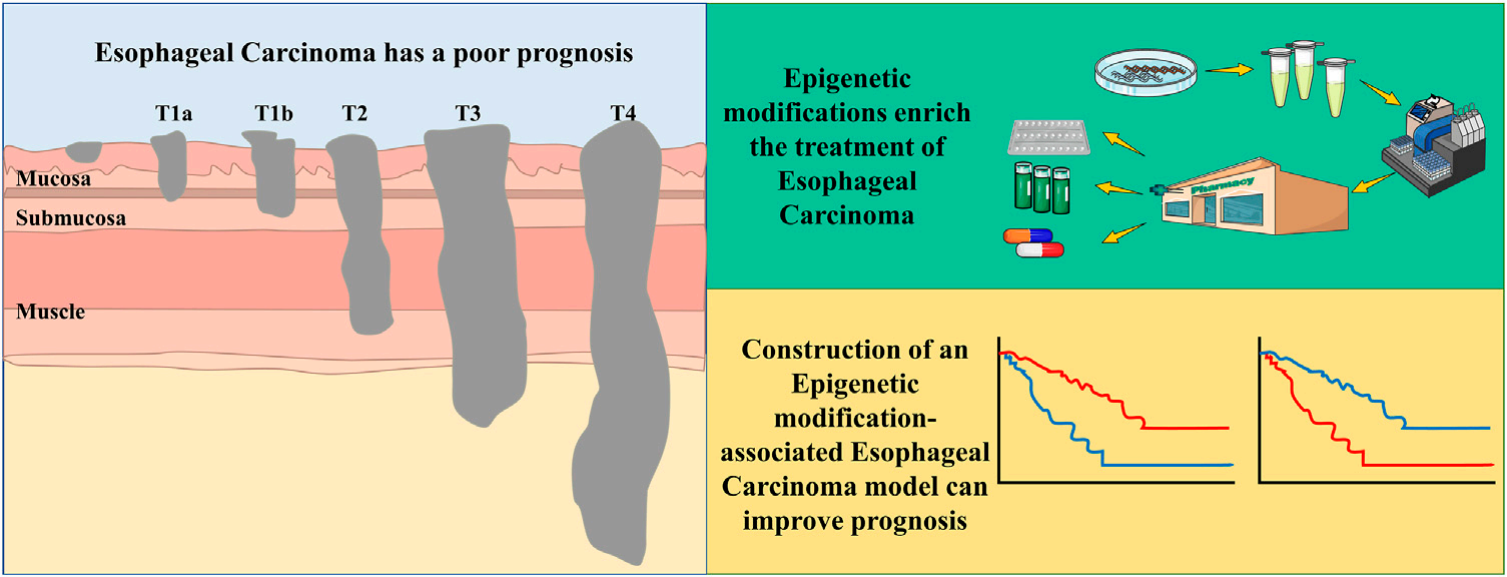
Esophageal Carcinoma being a cancer with poor prognosis, the addition of epigenetic mechanisms facilitates the early diagnosis, prognostic evaluation and therapeutic drug development aspects of the tumor.

Histone modifications mainly include methylation, acetylation, phosphorylation, adenylation, ubiquitination, glycosylation and the recently discovered lactation. Recently, researchers have proposed the use of global patterns of histone modifications as predictors of outcome in cancer patients. Zhao et al. performed a retrospective clinicopathological analysis of 97 patients with ESCC recovering from oesophagectomy and evaluated five histone modification markers, including H3K18Ac, H4K12Ac, H4R3diMe, H3 K4diMe and H3K27triMe. The obtained results revealed that low expression of H3K18Ac and H3K27triMe was associated with better prognosis in patients with ESCC, especially in early-stage patients (Tzao et al., 2009). In addition, Zhang et al. (2022) investigated five digestive cancers, including esophageal, gastric, hepatocellular, pancreatic and colorectal cancers, by systematically examining 13 HAT and 18 HDAC genes. As a result, histone acetylation was found to be a key regulatory molecule in digestive cancer. Moreover, H3K4me3, EP300, H3K9me3, H3K36me3 and H4K20me3 were all closely associated with the prognosis of esophageal cancer (Bi et al., 2019; Zhou et al., 2019; Ye et al., 2020). Furthermore, there are countless studies on the prognostic impact of non-coding RNAs on esophageal cancer. As confirmed by a recent multicentre prospective study, the detection of tsRNA in patients’ salivary exosomes can effectively distinguish patients with esophageal cancer from normal subjects with a specificity of 94.2% and can be used as a novel prognostic marker for esophageal cancer (Li et al., 2022). In addition, a prognostic model of esophageal adenocarcinoma risk based on miR-4521, miR-3682-3p and miR-1269a verified that miRNA target genes are significantly associated with immune infiltration, tumour microenvironment, cancer stemness properties and tumour mutational load in esophageal adenocarcinoma (Zhao J et al., 2021). Other prognostic factors regarding esophageal cancer include MALAT1, miR-21, miR-375 and miR-203 (Fu et al., 2014; Huang Y W et al., 2016; Wang Y et al., 2020) (Figure 3).

## 9 Discussion and prospects

Currently, the number of deaths due to cancer continues to increase with each passing year. Esophageal cancer, one of the most prevalent cancers of the digestive system, remains a challenge for mankind. Among the many cancer treatments available, the side effects of conventional treatment are mainly damaging to normal cells, which largely limits the application of some treatments. As research into molecular mechanisms progresses, researchers have been pursuing novel drugs with low toxicity and high specificity. Therefore, the development of new drugs with more targeting, better efficacy, fewer side effects and significantly lower cost will become a hot research topic in anticancer therapy. Based on this, the discovery of epigenetics provides an attractive target for the treatment of esophageal cancer and has received widespread attention from scholars at both national and international levels. DNA methylation is currently a relatively well-studied mechanism in epigenetics, but its mechanisms are still in the exploratory stage. Meanwhile, epigenetic modifications such as DNA methylation, histone modifications, non-coding RNAs and nucleosome localization are not independent of each other but often have synergistic effects, and their relationships need to be further investigated. This will help us to better understand and manipulate the epigenetic system and provide new perspectives for human disease prevention and control research.

Additionally, with the introduction of precision medicine services, new ways to prevent and diagnose cancer and accelerate the arrival of a new era of precision medicine are expected. With this aim, the construction of more non-coding RNA-related diagnostic and predictive models in esophageal cancer could help to provide new opportunities for the prevention and treatment of esophageal cancer (Zhao Y et al., 2021; Weidle and Birzele, 2022). Of note, some important breakthroughs have been made in the field of epigenetic regulation, including the development of epigenetic drugs. With a greater understanding of gene transcription, various epigenetic drugs have been developed and successfully applied in the treatment of cancer. Most notably, DNMTi- and HDACi-based drugs have been developed, which can be used not only alone but also in combination with antitumor drugs, improving their efficacy while largely reducing the toxic side effects of antitumor drugs. For example, the DNMTi 5-aza-2′-deoxycytidine combined with the antitumor effects of PD-1/PD-L1 provides a more effective immune response and clinical benefit for patients with esophageal cancer (Wu Y et al., 2020). The combination of 5-aza-2′-deoxycytidine with cisplatin, one of the conventional chemotherapeutic agents, also brings better therapeutic results (Cao et al., 2017). The drugs developed thus far, although showing promising advantages, still have toxic effects, such as bone marrow suppression and possible activation of oncogenes, which need further study.

As mentioned above, although a number of approved inhibitors targeting epigenetically modified enzymes have been developed and some are already in clinical trials, these advances suggest the great potential of epigenetic modifications in cancer therapy. However, as further thought and practice suggests, epigenetic modifications regulate cancer development not only in a single way but also in a synergistic way. The use of a single inhibitor alone is not sufficient to fundamentally alter the outcome of cancer patients, and the side effects cannot be avoided. Epigenetic modifications are reversible in nature, which may provide new ideas and targets for the prevention and treatment of esophageal cancer. Further research is needed to identify the exact efficacy and adverse effects of currently developed targeted drugs, and the existence of different pathogenesis mechanisms due to different regions or individual differences in esophageal cancer also requires further rational drug screening. In addition, the development of esophageal cancer is inextricably linked to environmental influences. All these factors add to the impediments to the translation of basic research into the clinical treatment of esophageal cancer. In fact, cancer treatment is a complex process, and it is difficult to achieve good results with one treatment alone; cancer treatment requires a combination of therapies to achieve the final victory in the battle against cancer.

Taken together, abnormal epigenetic alterations not only provide new opportunities for the diagnosis of tumours but also new strategies for their treatment. With further research on the relationship between epigenetics and esophageal cancer, epigenetic modifications are involved in the development of esophageal cancer through the regulation of various genes and signalling pathways, which also provides a more complete theoretical basis for the development of tumour epigenetic markers and antitumor epigenetic drugs. Based on this, the search for more precise molecular markers to serve the personalized characteristics of esophageal cancer and provide more reasonable treatment plans for each patient is the future direction of precision medicine, and the exciting performance of epigenetics in esophageal cancer is the cornerstone towards precision medicine.
